# The role of habitat mosaics on biological communities at hydrothermal vents and their periphery

**DOI:** 10.1038/s41598-026-39544-x

**Published:** 2026-02-18

**Authors:** Van Audenhaege Loïc, Sarrazin Jozée, Ramière Annah, Borremans Catherine, Marcillat Marin, Soto Vega Pedro Juan, Cannat Mathilde, Marticorena Julien, Colaço Ana, Matabos Marjolaine

**Affiliations:** 1 National Oceanography Centre, Southampton, UK; 2 Univ Brest, Ifremer, BEEP, 29280 Plouzané, France; 3 Institut de Physique du Globe de Paris, UMR 7154 CNRS, Université Paris Cité, Paris, France; 4 ABYSSA, French Company for the Deep-Ocean Exploration, Anglet, France; 5 University of the Azores, Institute of Marine Sciences–OKEANOS, Horta, Portugal

**Keywords:** Biodiversity, Ecology

## Abstract

Hydrothermal vent fields harbour a heterogeneous seascape owing to complex topography and vent fluid emissions, including in peripheral areas beyond active venting. At the Lucky Strike hydrothermal vent field, a remotely operated vehicle was used to acquire two hectares of seabed imagery to evaluate the role of environmental conditions on the structure and distribution of benthic communities. Our analyses revealed that large and mature edifices are potential keystone structures supporting hotspots of vent specialists (e.g., *Bathymodiolus azoricus*) and associated fauna (e.g., Zoanthidae). Higher densities of mobile bathyal shrimps were found in slabs adjacent to active venting areas. Communities occupying areas with lower hydrothermal exposure displayed significantly higher diversity than areas located closer (≤ 40 m) to vent emissions. Between 40 and 120 m from active sites, community diversity was greatest on hard substrata dominated by sessile organisms—such as octocorals, Cladorhizid and Hexactinellid sponges—in greater densities when these substrates were composed of basalt with complex topography. While the gradient in hydrothermal exposure creates a spatial zonation of benthic communities, abundance and diversity patterns remain locally conditioned by seabed topography and substratum hardness. This interplay fosters a patchwork of faunal communities at the decametre scale. These results underscore the complexity of designing surveys encompassing the diversity of habitat conditions within the deep hydrothermal seascape. Although historically overlooked, the diversity of distinct benthic communities beyond venting zones emphasises the ecological significance and vulnerability of areas that could be targeted by mining.

**Supplementary Information:**

The online version contains supplementary material available at 10.1038/s41598-026-39544-x.

## Introduction

Numerous studies have demonstrated the importance of the physico-chemical gradient created by the mixing of the hot vent fluids and cold seawater to create a myriad of habitats in hydrothermal ecosystems. The fauna occupies these habitats depending on their trophic need and tolerance to vent exposure, resulting in spatial mosaic patterns of assemblages^1–3^. Typically, endemic vent species live close to fluid emissions emitted on active sulphide structures or through fissures on the seafloor^4,5^. Theoretically, as the distance to the vent fluid increases, conditions should become more favourable for the colonization of an outer, less adapted fauna, composed of non-vent species^6,7^. The few studies focusing on non-active areas showed that peripheral megafauna display differences in composition and abundance compared to the observed regional faunal communities^8^, as a response to hydrothermal primary productivity, through the export of prey, organic material and vent particles swept away by enhanced advective currents^9,10^.

In addition, the nature of the substratum appears to influence habitat suitability for colonisation of sessile organisms^11,12^. Hydrothermal vent fields typically harbour various geological features that create a complex spatial arrangement of venting sites and inactive areas with a diverse range of substratum types^13,14^. The interaction of these environmental variables further contributes to enhance the heterogeneity and patchiness of benthic habitats. Similarly to what is observed on vent edifices^15^, the complex combination of environmental factors in peripheral areas should lead to mosaic distribution patterns of megafaunal species over scales of hundred metres to kilometre as suggested in the Pacific and Indian oceans^16,17^. These particular areas matter from a conservation perspective, as they are typically associated with enhanced biodiversity^18^. However, monitoring of seascape mosaics requires survey systematics that capture the spatial dependencies at which different processes occur and interact^19^. The development of deep-sea observatories, and their regular maintenance cruises, contribute to increase the time allocated to the standardised acquisition of optical images, thus facilitating the systematic collection of inter-comparable seabed imagery^20^.

Since 2010, the Lucky Strike hydrothermal vent field, located on the Mid-Atlantic Ridge, hosts the EMSO-Azores observatory, which lies within a Portuguese marine protected area. Lucky Strike is a basalt-hosted hydrothermal field located on top of an axial volcano that displays a fossil lava lake surrounded by three volcanic cones^21,22^. At the field-scale, several factors including the distribution of vent emissions, physico-chemical conditions, nature of the substratum and hydrodynamics contribute to increase habitat heterogeneity^13,23,24^. While an extensive ecological knowledge on vent fauna at active Lucky Strike sites is available, mapping of peripheral megafaunal communities at this vent field has never been done. Interestingly, a recent environmental DNA (eDNA) metabarcoding approach highlighted strong differentiation in biological communities between active, inactive and peripheral habitats, with a much higher diversity in peripheral and inactive zones^25^.

Hereby, we take advantage of the extensive mapping effort deployed at Lucky Strike in 2018 and the systematic sampling design developed (1) to map the hectare-scale distribution of vent and peripheral communities in two extensive seafloor areas around active vents and (2) to assess the role of structuring factors (i.e., fluid composition, hydrothermal exposure, substratum) and their interactions on the distribution of faunal communities. Considering the potential for habitat heterogeneity and associated community mosaics, this ecological survey serves as a basis to develop a deeper understanding on biodiversity patterns. This ecological understanding is particularly timely as it can inform regulatory decisions and guide monitoring strategies designed to preserve these environments, particularly in a contemporary context where mining of seafloor massive sulphide deposits is gaining interest^26^.

## Materials and methods

**Fig. 1:**
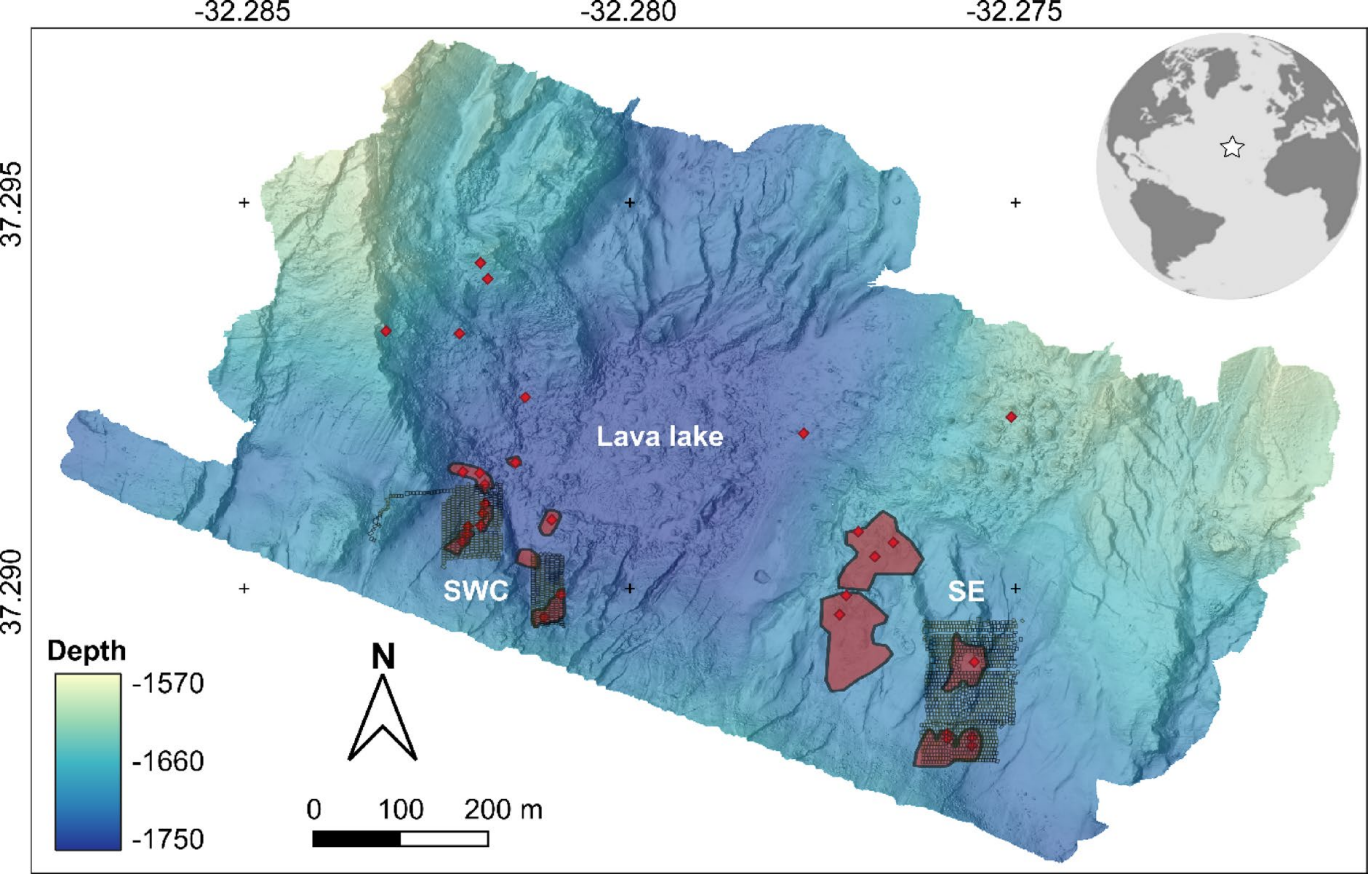
Bathymetric map of the Lucky Strike hydrothermal vent field (WGS84), located on the Mid-Atlantic Ridge. Imprints of seabed images are delimited with small black polygons. Hydrothermal edifices and the closest venting areas to this study are indicated by red points and polygons, respectively. The location of the different regions sampled is denoted with bold white text, with the south-west central area (SWC) encompassing the Sapin, White Castle, South Crystal, Crystal and Pico active vent edifices, and the south-east area (SE) containing the Montsegur, Cimendef and Eiffel Tower vent edifices. The upper right inset locates Lucky Strike in the Atlantic Ocean.

The image set was collected during the MoMARSAT 2018 cruise using the Remotely Operated Vehicle (ROV) *Victor6000* deployed from the research vessel *L’Atalante*. Transect waypoints were positioned 1.8 m apart from each other to encompass rectangular areas around active vent edifices. Two regions of the vent field, known to represent different chemical domains^23^, were explored: the south-west central (SWC) and south-east (SE) areas (Fig. 1). These regions differ in terms of the geological setting of the vent sites, the SWC area located along fissures next to the fossil lava lake, while the SE area is located in a topographically higher domain that has been uplifted by normal faults^22^. In terms of the fluid chemistry of the end member black smoker, fluids from the SWC area have higher chlorine contents, while fluids from the SE area have lower Na, Ca, Br, K, Mn, Sr and LI contents. These lower concentrations in the SE are consistent with their lower chlorinity, indicative of a larger vapor to brine proportion than at the SWC sites^23^. During the image acquisition, *Victor6000* was flown at a constant speed (~ 0.2 m s^− 1^) and altitude of 4 m above the seabed, and its downward-looking high-definition OTUS camera^27^ was set to automatically capture one .JPG image (4000 × 6000 pixels) every 3 s. Recordings of an ultra-short baseline system (USBL), of a doppler velocity logger (DVL) and of a photonic inertial navigation system (PHINS) were combined to optimise the accuracy of the ROV positioning on the seafloor.

The first step consisted in the screening of all images to manually discard the low-quality ones (i.e., blurred or obscured). A set of non-overlapping images (*n* = 1626) was produced after reprojection of the image imprint using an orthophoto tool involving intrinsic parameters of the OTUS camera and extrinsic parameters of the ROV e.g., altitude, pitch, roll, heading and X-Y navigation coordinates (*MATISSE*^28^ v.1.4.0). Using the default settings of the *MATISSE* pre-processing tool, we applied contrast enhancement and colour correction on the subset of non-overlapping images to correct for underwater light dissipation and non-uniform illumination. In addition, the *MATISSE* orthophoto tool was used to create georeferenced scaled .tiff images to estimate their areal imprint from which image resolution was estimated at 0.8 ± 0.1 mm px^−1^ (µ ± SD).

The non-overlapping images were uploaded on *BIIGLE* for manual annotations of biological features^29^. Vent assemblages (i.e., aggregation of > 5 individuals) and microbial mat edges were detoured with polygons. Non-aggregating vent and non-vent fauna were annotated individually. All annotations were re-evaluated using the *BIIGLE* largo tool to gather organisms with similar morphology in groups identified to the lowest taxonomical level possible, hereafter ‘morphotypes’, following image-based specimen classification of Horton et al.^30^. Topographic complexity is expected in hydrothermal vent fields^13^, which challenges body length measurement with 2D images. To avoid potential uncontrolled bias in community composition among habitats, we did not apply the 10 mm size cut-off, typically used to retain megafaunal annotation. Instead, we annotated all visible fauna and retained morphotypes with a body length that confidently fell into the megafaunal size class. Due to differences in annotation methodology between vent assemblage/microbial mat and individualised organisms, abundances were measured with different units: areal cover and counts, respectively.

Each image was assigned a set of values representing environmental variables: (1) a site factor as a proxy for distinct chemical domain^23^, (2) a distance factor from closest venting emission as a proxy for hydrothermal exposure and (3) a seabed factor –of lithology– as a proxy for substratum properties (Table 1). Images were discriminated between the SE and SWC sites to produce two areas (Fig. 1). Hydrothermal vent exposure was approximated using the distance from the image barycentre to the closest venting area^12^ (Fig. 1). Venting areas were delimited manually as polygons in QGIS (v.3.34), using the presence of hydrothermal minerals and vent faunal assemblages observed from this study and from image mosaics of precedent surveys^13^. Levels of hydrothermal exposure were factorised as follows: within venting area, high [0, 20 m from venting area], medium [20, 40 m] and low [40, 120 m]. Finally, the dominant seabed lithology (representing > 50% of image areal coverage) was annotated in *BIIGLE* (i.e., sulphide deposits, indurated volcaniclastic deposits, hereafter ‘slab’, basalt or volcaniclastic sediment). No images contained three or more substrata each occupying less than 50% of the image area. Substratum labelling was expedited using a deep learning algorithm followed by visual validation^31^.

Sampling effort completeness was assessed using sample-size-based rarefaction curves with an ‘image’ (µ ± SD: 14.35 ± 3.84 m^2^) as the sample-based data and presence-absence community data, using the *iNEXT* package (v.3.0.1)^32^. Taxonomic diversity was computed with sample-size-based rarefaction curves with individual morphospecies counted as the sampling unit. Rarefaction curves were extrapolated at 3000 images and individuals to reach a representative sampling effort since approaching the theoretical asymptote of the curve. All rarefaction curves were interpolated and extrapolated with a Hill number q = 0 and q = 1, estimating the taxonomic richness and Shannon diversity, respectively. Bootstrap 95% confidence intervals were computed for interpolated and extrapolated rarefaction curves^33^. As a measure of community uniqueness, local contribution to β-diversity (LCBD) was computed among substratum and vent exposure factors, using the sum of squares of Hellinger-transformed total morphospecies abundance randomly subsampled to an equal seabed area per environmental factor (*adespatial* package)^34,35^. LCBD indices range between 0 and 1 being the maximum contribution to β-diversity. Holm-corrected significance of LCBD indices were assessed with random independent permutations of morphotype abundances (n = 999)^34^.

Faunal densities were compared between each environmental factor. Since one image is not a statistically representative unit of sampling, images were pooled into tiles accounting for 120–140 m^2^ following Benoist et al.^19^. Images were pooled randomly to degrade any spatial autocorrelation that could contribute to the residual variability^19^. Bounds of the 95% confidence interval were computed using a Student’s t-Test. Pairwise differences in densities were tested using a Tukey *post hoc* test.

Multivariate analyses were also performed by pooling neighbouring images into tiles of 120–140 m^2^. Compared to univariate density analyses, we decided not to degrade spatial autocorrelation to capture community transition occurring in potential ecotones. While site remained binary, substratum and vent exposure were aggregated considering their relative areal proportion within a tile. A redundancy analysis (RDA; scaling = 1) was computed on Hellinger-transformed faunal densities to disentangle the relative contribution of environmental drivers on the variance of the community composition and tested with a permutation-based ANOVA-like test (perm. = 999, function *anova*, *vegan* package, v.2.6.4)^36^. Only significant RDA axes were retained for graphical display (function *triplo.rda*)^37^.

The ichthyofauna was excluded from biodiversity and abundance-based analyses because of their high mobility enabling habitat occupancy beyond the scale of this study. Furthermore, biodiversity and abundance-based analyses did not include vent assemblage and microbial mats because of unit differences. All analyses were performed on R (v.4.3.2). Significance levels were computed at α = 0.05.

## Results

### Habitat description

The *Victor6000* ROV required 19 h to survey 23,336 m^2^ of seabed with the collection of 1,626 non-overlapping images (Table 1). Although sampling effort was expectedly unbalanced among factors, at least 18 tiles were acquired per factor of seabed (Table 1).

**Table 1:** Image footprint and image/tile number associated with environmental factors.

Variable	Factor	Area [m^2^]	Image #	Tile #
Site	South-east (SE)	14,593	1020	110
	South-west central (SWC)	8743	606	67
Lithology	Basalt	3061	201	24
	Slab	12,917	910	98
	Sulphide deposits	3927	274	30
	Volcaniclastic sediment	3431	241	26
Hydrothermal exposure	Within	4972	347	38
	High	9638	685	73
	Medium	6451	446	49
	Low	2275	148	18

**Fig. 2:**
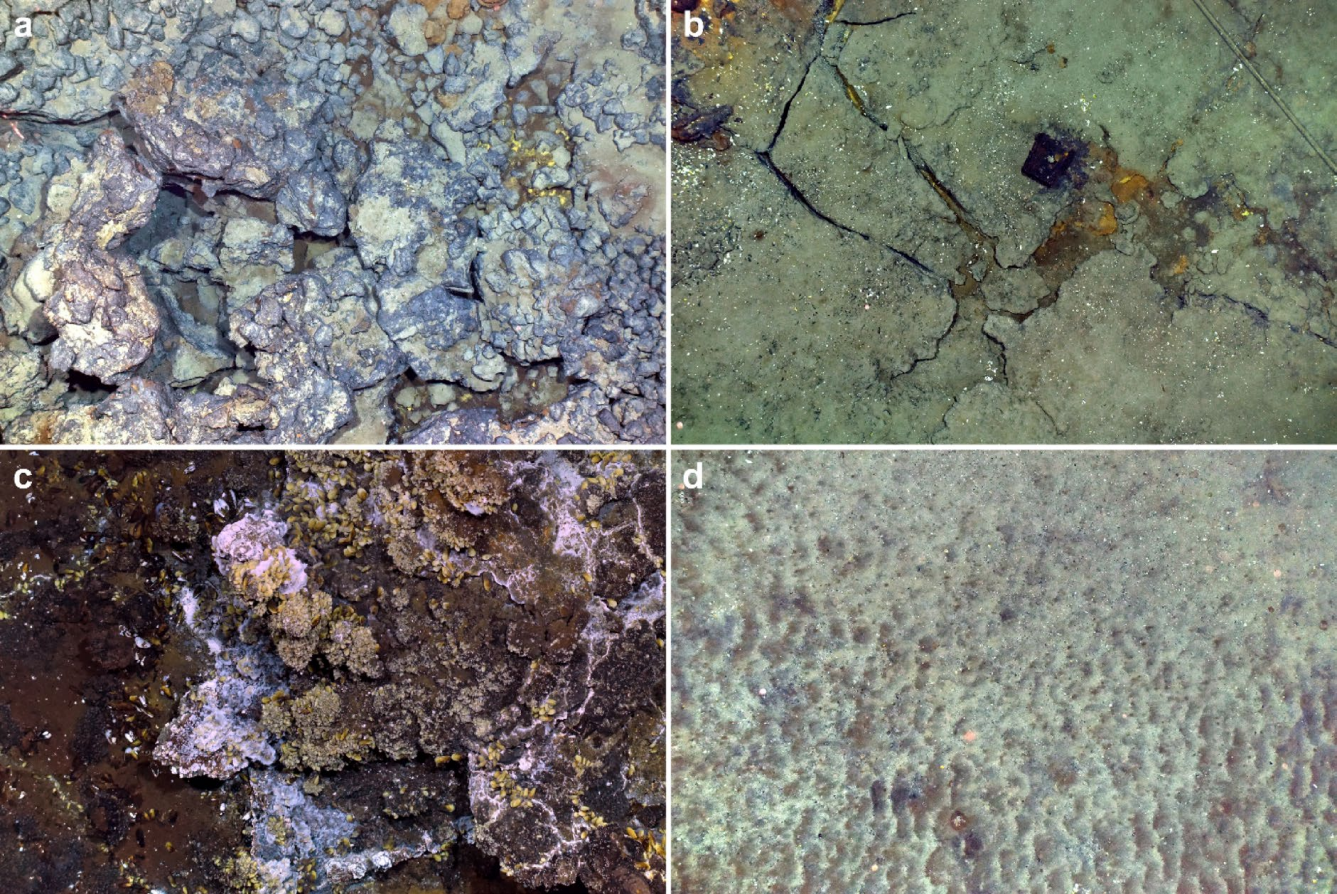
Substrata observed at the Lucky Strike vent field: (**a**) basalt displaying a heterogeneous topography with the presence of scree rubbles, (**b**) slab with the presence of brown iron-oxidising microbial mats, (**c**) sulphide deposits covered by vent mussel assemblages of *Bathymodiolus azoricus* and white sulphur-oxidising microbial mats, (**d**) volcaniclastic sediment with ripples. See geology definitions of substrata in^14^.

The four types of substrata were encountered at both SWC and SE sites, predominantly including hard substratum, with higher topographic complexity in basalts compared to sulphide deposits and slab (Table 1; Fig. 2a–c). To a lesser extent, areas of softer substratum were also observed (i.e. volcaniclastic sediment; Table 1; Fig. 2d). Except for the slab which extended over large and continuous areas, substratum formed patches of ~ 10 to 20 m in width and length (Supplementary Fig. 1).

### Coverage of vent assemblages

The SE site hosted a four-fold larger coverage of vent-endemic mussel *Bathymodiolus azoricus* (80.3 m^2^) compared to the SWC (18.3 m^2^; Table 2; Fig. 2c). *Bathymodiolus azoricus* assemblages predominated over vent edifices and their surroundings, in cracks (Supplementary Fig. 1). Dense aggregations of undescribed Zoanthidae gen. indet. were found exclusively at the periphery of the mussel assemblages on the Eiffel Tower and Montsegur edifices in the SE (27.6 m^2^; Table 2). Observable microbial communities were characterised by white or orange mats, corresponding to sulphur or iron oxidising microorganisms, respectively (Table 2; Fig. 2b, c). Sulphur oxidising mats covered 67.8 m^2^ and 25.3 m^2^ in the SE and SWC sites, respectively. Iron oxidising mats covered 166.9 m^2^ and 171.1 m^2^ in the SE and SWC sites, respectively.

**Table 2:** Distribution of areal coverage [m^2^] of vent faunal assemblages and microbial mats.

Habitat factor	*B. azoricus*	Zoanthidae gen. indet.	Sulphur oxidising mat	Iron oxidising mat
Site				
South-east	80.3	27.6	100.7	166.9
South-west central	18.3	0.0	30.2	171.1
Substratum				
Basalt	0.3	0.0	0.3	8.2
Slab	0.4	0.7	7.1	225.1
Sulphide deposits	97.9	26.9	123.5	96.8
Volcaniclastic sediment	0.0	0.0	0.0	7.9
Hydrothermal exposure				
Within	97.2	26.9	125.4	122.4
High	1.4	0.73	5.4	118.2
Medium	0.0	0.0	0.1	88.6
Low	0.0	0.0	0.0	19.8

### Community composition

**Fig. 3:**
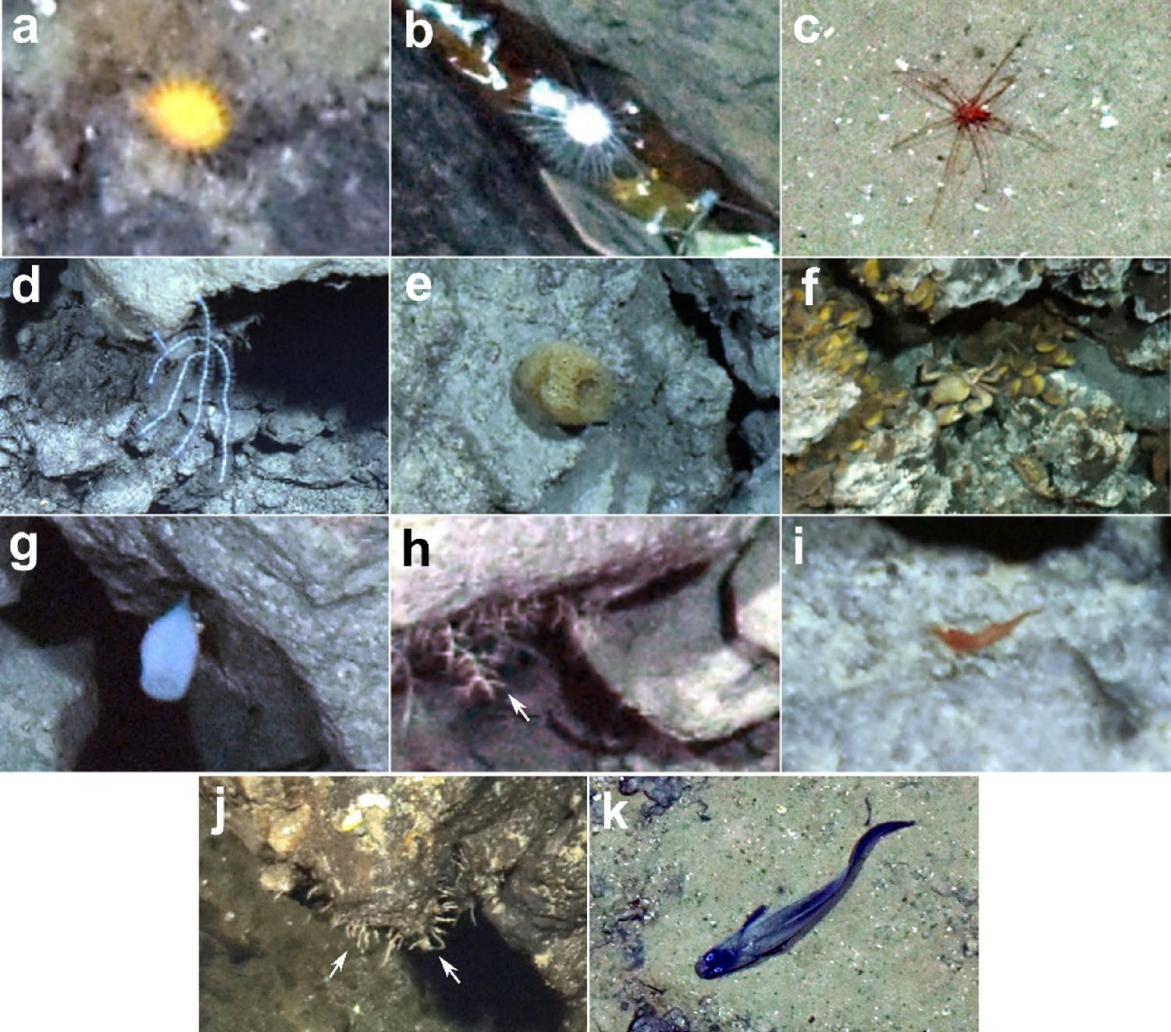
The nine most abundant morphospecies observed at Lucky Strike (≥ 10 individuals). (**a**) Actiniaria fam. indet. (*n* = 10) (**b**) Echinoidea order indet. (*n* = 11) (**c**) Pycnogonida gen. indet. (*n* = 18) (**d**) Blue Octocorallia order indet. (*n* = 35) (**e**) Demospongiae order indet. (*n* = 49) (**f**) *Segonzacia mesatlantica* (*n* = 121) in assemblage of *Bathymodiolus azoricus* (**g**) Hexactinellid order indet. (*n* = 278) (**h**) Arborescent Cladorhizidae gen. indet. (*n* = 348) (**i**) Decapoda fam. indet. shrimp (*n* = 1,713) (**j**) Arboramminid foraminifera sp. aff. *Luffammina atlantica* (*n* = 834) (**k**). *Cataetyx laticeps* fish (*n* = 62).

A total of 3487 annotations of individual organisms were performed. Thirty-three morphotypes of metazoan invertebrates were detected. They belonged to the phylum Annelida (2 morphotypes), Arthropoda (6), Mollusca (1), Echinodermata (5), Cnidaria (16) and Porifera (3). Eleven morphotypes corresponded to singleton observations and twelve occurred between 2 and 5 times. Nine morphotypes of metazoan invertebrates had an abundance greater than 10 individuals (Fig. 3a–i). Dominant morphotypes were found with abundance > 250 individuals and included sessile porifera belonging to the Hexactinellida class and Cladorizhidae family and decapod shrimps (Fig. 3g–i). One protozoan morphotype of the phylum Foraminifera was detected (Fig. 3j).

Although excluded from analyses, the fish community included 10 morphotypes, dominated by *Cataetyx laticeps* representing 46% of the 136 fish occurrences (Fig. 3k). Other observations consisted in other morphotypes typically found at Lucky Strike^38,39^ including Gadiformes cods fam. indet., identified for some observations as *Gaidropsarus mauli* and *Nezumia sclerorhynchus*, eel-like specimens, *Polyacanthonotus rissoanus* and *Synaphobranchus kaupii*, and Chondrichtyes, with *Hydrolagus pallidus* and sporadic observations of Elasmobranchii fam. indet. specimens.

## Diversity

**Fig. 4:**
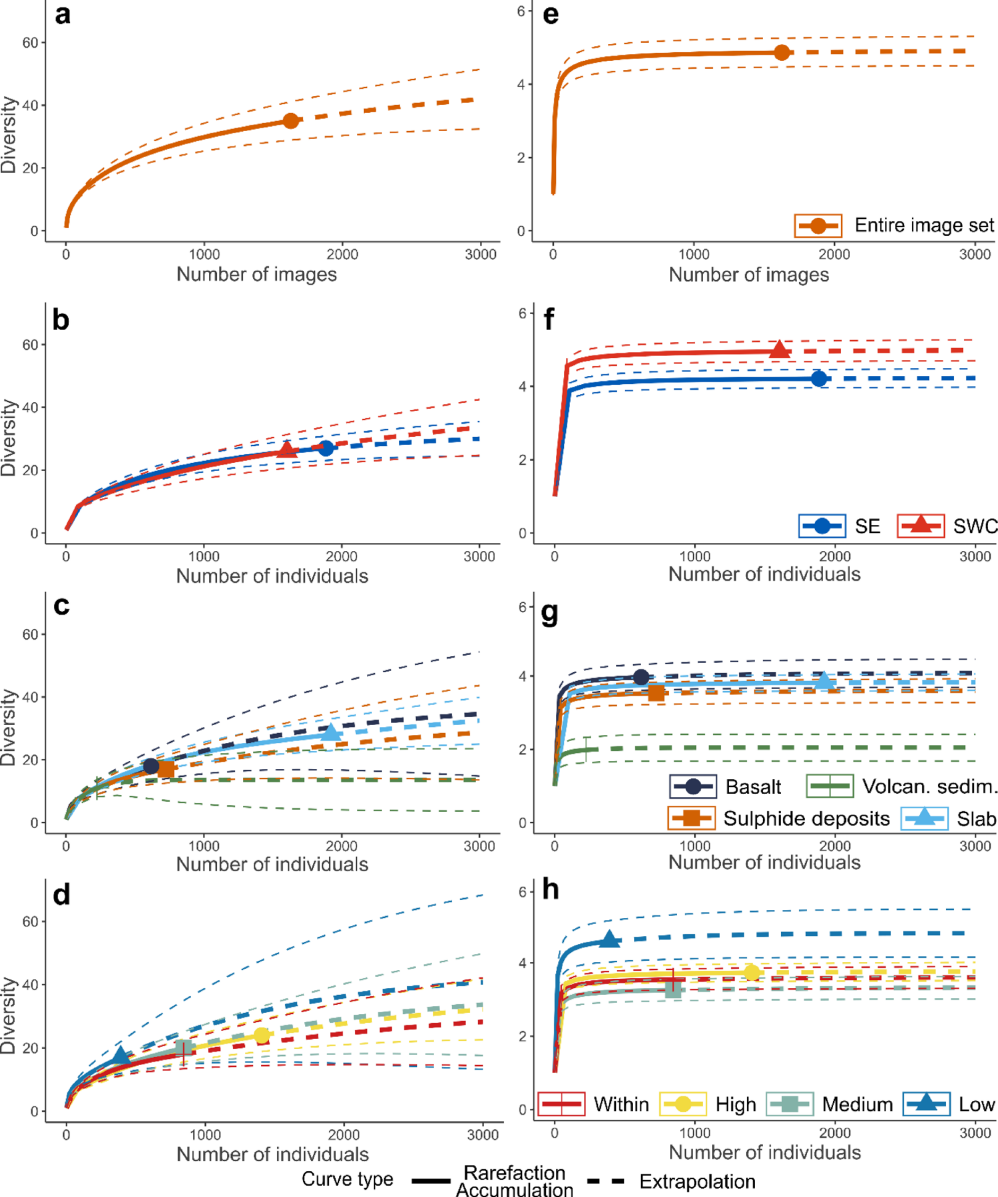
Image-based accumulation and individual-based rarefaction curves considering two Hill’s numbers: panels (**a**–**d**), q = 0 (i.e., morphotypes richness); and (**e**–**h**), q = 1 (i.e., Shannon index). Rarefaction curves were computed for the entire image set (**a**–**e**), by site (**b**–**f**), substrata (**c**–**g**) and hydrothermal exposure (**d**–**h**). A single image represents on average 14.35 m^2^. Extrapolation was made at 3000 images and individuals (thick dash lines). Significant differences are delineated with 95% confidence intervals (thin dash lines; perm. = 999).

The image-based accumulation curve for q = 0 suggested the sufficiency of the sampling effort with the curve approaching an asymptotic richness value of 42 ± 8 morphospecies (Fig. 4a). Individual-based morphospecies rarefaction curves showed no significant differences in richness (q = 0) among communities discriminated by site and hydrothermal exposure (Fig. 4b–d). Regarding substratum type, extrapolation at 3000 individuals suggested that richness (q = 0) reaches higher values in slab-associated communities than in volcaniclastic sediment (Fig. 4c). The image-based accumulation curve for q = 1 suggested the sufficiency of the sampling effort with the curve reaching an asymptotic Shannon index of 4.9 ± 0.3 (Fig. 4e). Differences in community diversity became more apparent with a higher Hill’s number (q = 1), pointing out differences in community evenness (Fig. 4f–h). Significant higher diversity (q = 1) was observed in the south-west central site (SWC, Fig. 4f). Volcaniclastic sediment exhibited significant lower diversity (q = 1) than the rest of the substratum types (Fig. 4g). Communities occupying areas with lower hydrothermal exposure displayed significantly higher diversity (q = 1) than areas located closer (≤ 40 m) to vent emissions (Fig. 4h).

Local contribution to β-diversity was higher and significant for communities located within venting areas (LCBD = 0.56, p adj.=0.02), followed by low (LCBD = 0.20), medium (0.15), and high (0.08) hydrothermal exposure, although not significant. Local contribution to β-diversity was higher and significant for communities located over sulphide deposits (LCBD = 0.51, p adj.=0.02). Local contribution to β-diversity was not significant for basalt (LCBD = 0.24), volcaniclastic sediments (0.19) and slab (0.06).

### Densities

**Fig. 5:**
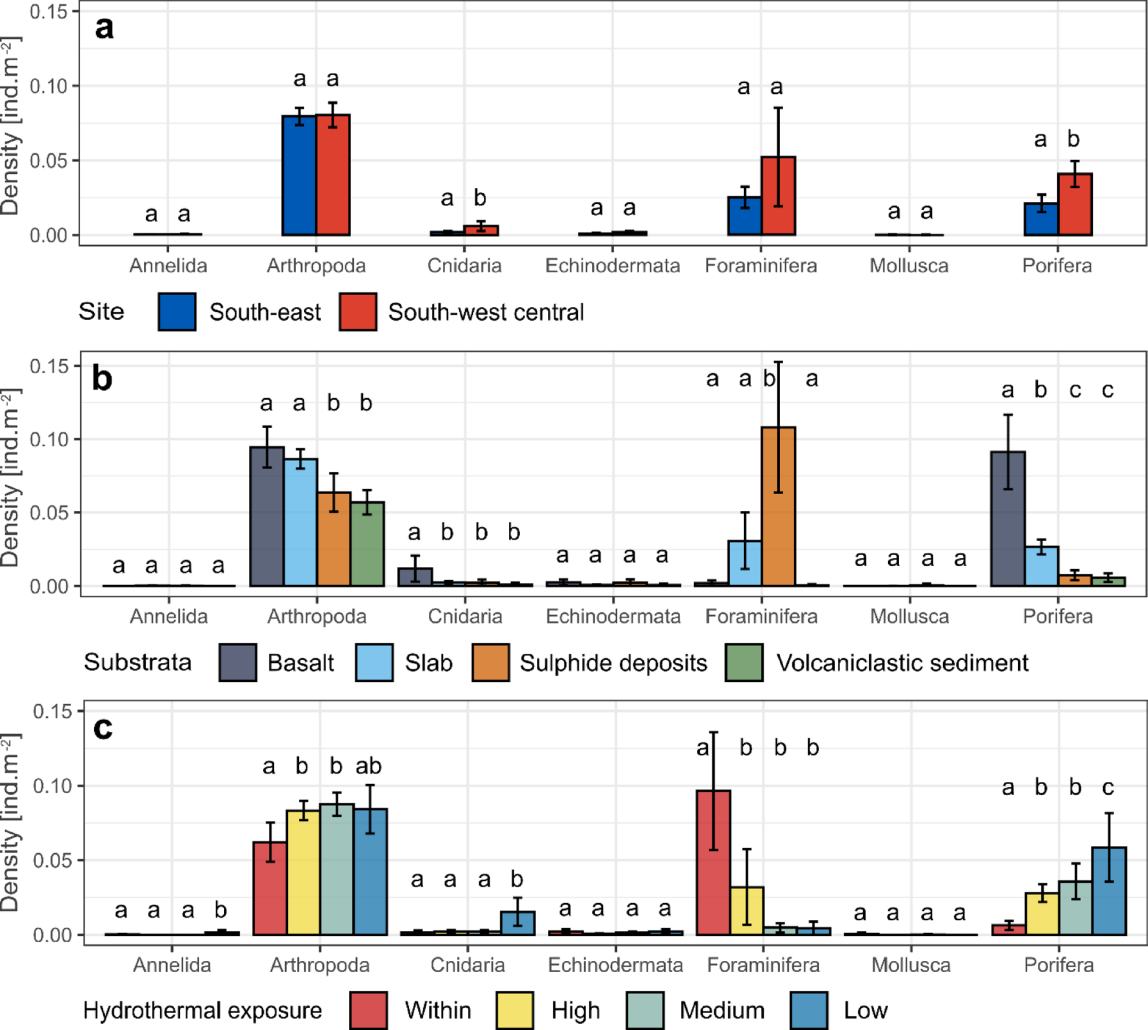
Barplots of phylum densities computed from tiles in which seabed images were pooled randomly (see Table 2). Panels present independent results of the effect of (**a**) site, (**b**) substratum and (**c**) hydrothermal exposure. Different letters on top of barplots indicate significant density differences using a Tukey *post hoc* test (see Supplementary Table 1). Whiskers indicate bounds of the 95% confidence interval computed with a Student’s t-test.

Excluding foraminifera, metazoan densities reached on average 0.12 ind.m^− 2^ with a high degree of variability among images, ranging from 0 to 2.17 ind.m^−2^ with a standard deviation of 0.17 ind.m^−2^. Phylum-based densities pointed out significantly higher densities of Porifera and Cnidaria in the SWC area, consisting mainly of anemones and soft corals (Fig. 5a). Higher densities of Arthropoda were found on slab and basaltic areas compared to sulphide deposits and volcaniclastic sediments (Fig. 5b; see Supplementary Table 1 for details on statistical results). Furthermore, Arthropoda had significantly lower densities in active venting areas (Fig. 5c). Cnidaria had significantly higher densities on basalt compared to the other substrata. Cnidaria also harboured higher densities at low hydrothermal exposure (Fig. 5c). Porifera densities were higher on basalt, followed by slab (Fig. 5b), with significantly lower densities within active venting areas, and higher in lower hydrothermal exposure, compared to medium and high venting exposures (Fig. 5c). Foraminifera were significantly denser on sulphide deposits than any other substratum, and to a lower extent on slab than on volcaniclastic sediment (Fig. 5b). Similarly, they clustered more densely within active areas (Fig. 5c). Some phyla like Annelida, Echinodermata and Mollusca displayed low densities (< 0.01 ind.m^2^) and no significant remarkable aggregation were observed, except for Annelida that were significantly denser (10^−3^ ind.m^−2^) under low exposure conditions (Fig. 5c).

### Drivers of community composition

**Fig. 6:**
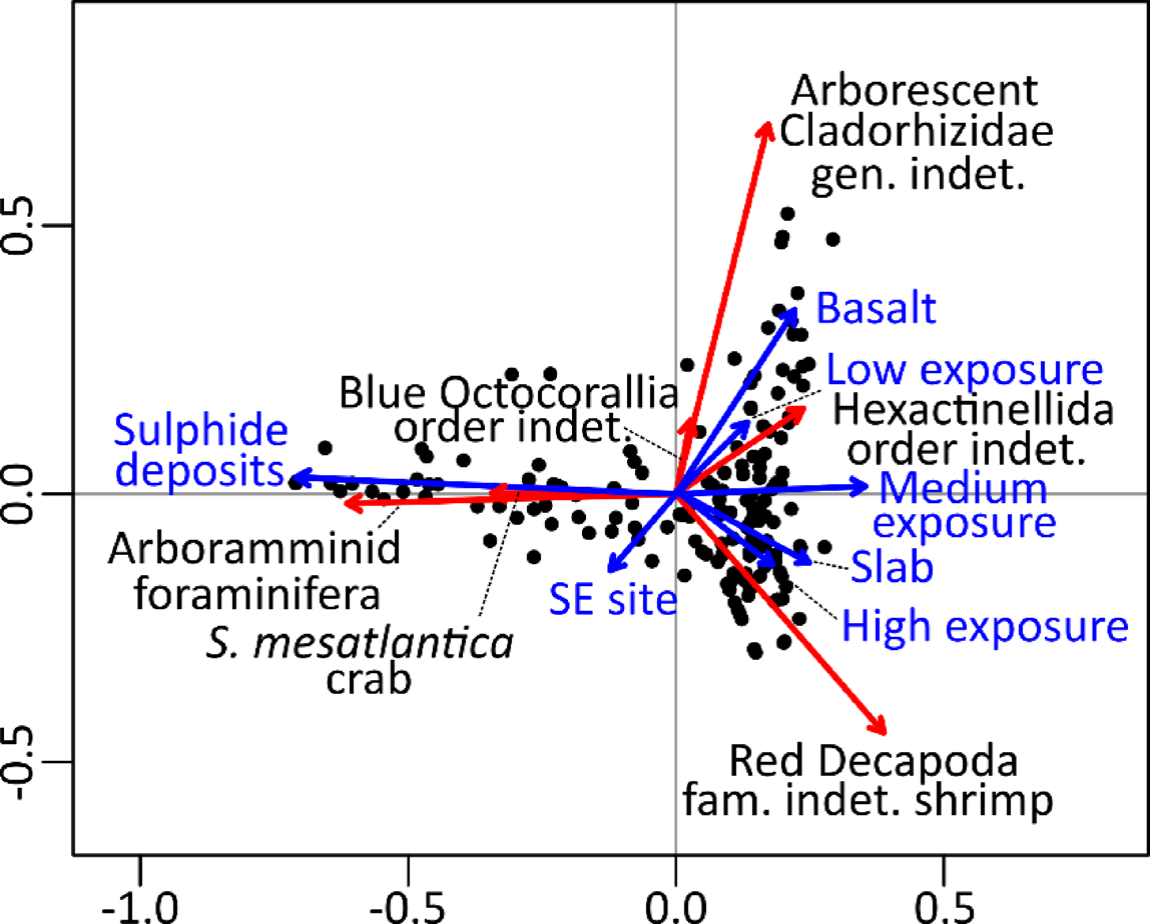
Multivariate analyses to assess the influence of environmental drivers on community composition. Biplot of the first two axes derived from a redundancy analysis (RDA), explaining 21.3% and 6.8% of the variance, respectively. Explanatory and response variables were coloured in blue and red, respectively. Only morphotypes with a goodness of fit ≥ 0.1 were drawn for plot clarity.

The first two axes of the RDA performed on the 184 tiles covering in average 126.8 m^2^, explained together 28.1% of the variance (Fig. 6a). The first axis of the RDA was significant (F_1,176_=54.4, *p* = 0.001) and separated *S. mesatlantica* and Arboramminid foraminifera, that dominated sulphide deposit habitats, from the rest of the morphotypes populating areas outside hydrothermal venting areas (Fig. 6a). The second RDA axis was also significant (F_1,176_ = 17.5, *p* = 0.001) and depicted a spatial zonation among substrata interacting with vent exposure. High densities of red bathyal shrimps were found in high exposure and slab areas (Fig. 6a) and were segregated from Hexactinellid order indet. associated with medium exposure areas. In lower venting areas, blue Octocorallia order indet., Cladorhizidae gen. indet. and Hexactinellida order indet. were found in high densities when associated to the basaltic seabed (Fig. 6). Excluding volcaniclastic sediment as a covariate, all substrata were significant including basalt (F_1,176_ = 17.4, *p* = 0.001), sulphide deposits (F_1,176_ = 48.7, *p* = 0.001) and slab (F_1,176_ = 4.2, *p* = 0.004). Excluding areas within vent emission as a covariate, significance was found for high exposure (F_1,176_ = 2.9, *p* = 0.021), but not for medium (F_1,176_ = 2.1, *p* = 0.069) and low (F_1,176_ = 2.7, *p* = 0.073) exposures. The influence of the site was significant (F_1,176_ = 2.4, *p* = 0.038), with lower densities of basalt-hosted organisms found in the SE area. No clear clustering was observed which depicts the gradient in morphotypes composition along the levels of hydrothermal exposure.

## Discussion

Using an extensive seabed imaging effort, this study has successfully described the distribution patterns of the benthic fauna in venting and peripheral areas. We attempted to further disentangle the relative contribution of habitat variables on the community composition. Firstly, we detected the presence of potential keystone structures for vent assemblages associated with distinct sites. Secondly, we highlighted how the interaction between hydrothermal exposure and substratum types influences community structure up to 120 m from active venting areas.

### Potential for keystone structures

The vent endemic mussel *Bathymodiolus azoricus* was found in both sites located in distinct sites^23^, albeit with different coverages; the absolute cover at SWC being four times smaller than at SE (80 m^2^). When making it relative to the proportion of imaged seabed, the mussel cover at SWC remains 2.5 times smaller than at SE. Furthermore, the cover measurements at the SE area were underestimated since the vertical walls of the Eiffel Tower edifice were not imaged (Supplementary Fig. 1), despite hosting extensive mussel assemblages (270 m^2^;^40^). The greater cover observed in the SE may be linked to variations in the fluid composition between the two sites and their distinct chemical domains^23^, which affects physicochemical conditions such as differences in metal speciation and partitioning^41,42^. In turn, this can influence the physiology and distribution of *B. azoricus*^43–45^. Indeed, this species benefits from a dual symbiosis with sulphide- and methane-oxidizing bacteria^46^ which can vary according to local environmental conditions. Variations in sulphur and methane concentrations could therefore explain variations in mussel coverage^47^, as these mussels appear to rely more heavily on their methanotrophic endosymbionts as they become larger^48^. At the SWC area, this could potentially result in a more restricted niche breadth of conditions favourable to *B. azoricus* and its associated symbionts, resulting in lower mussel cover compared to the SE area.

Alternatively, the development of mussel assemblages might be facilitated by the geomorphology of vent edifices, and particularly by the presence of complex structures as the Eiffel Tower edifice^40^. At the SWC area, edifices lacking a sulphide talus extend over smaller surfaces (i.e. < 20 m^2^) and harbour a few black smokers on their summits^13,14^. These edifices are typically considered young, friable and ‘immature’^15,49^. As they consolidate and, as the talus expands in size and width, ‘mature’ sulphide edifices will harbour more diffuse outflows at their base, since permeability decreases on the top of the edifice^49^, like in the SE area. There, edifices like Montsegur and Eiffel Tower form larger structures (i.e. 50–250 m^2^) with abundant diffuse outflows extending at their bases and periphery^13,14,40,50^. Therefore, these consolidated edifices provide larger surfaces exposed to the vent fluid rising from cracks and diffuse outflows scattered at the edifice base and bathing vent-obligate species on the edifice walls with the buoyant vent fluid^24,51^. The large area of seabed suitable for vent fauna colonisation on the talus and their peripheries thus considerably increases the carrying capacity of these edifices, which explains the presence of vent assemblage ‘hotspots’ at the SE area of Lucky Strike. Hence, by providing an extended access to resources and a stable and consolidated habitat for colonisation, we therefore hypothesise that such edifices could be specifically considered ‘keystone structure’ for the vent fauna^52^. Even, the low regime of disturbance at these edifices has been linked to the climax stage of this extensive cover of *B. azoricus*^40^. As large and stable populations are typical descriptors of source populations of larvae^53^, they hold potential to significantly contribute to the maintenance of the metapopulation at the larger scale of the northern MAR. Considering this, conservation practices should draw a particular focus on the preservation of keystone structures concentrating endemic communities, especially when intending to mitigate ecological disruption of anthropogenic pressures^54^.

### Assemblage mosaics

Vent-endemic morphotypes were found as far as 20 m away from venting areas. This is the case of the foraminifera *Luffamina atlantica*, a genus typically observed within venting emission areas such as at the Rainbow vent field in the MAR^55^, and the Bythograeid crab *Segonzacia mesatlantica*, typically associated with mussel bed assemblages^56^. Furthermore, dense aggregations of undescribed Zoanthidae gen. indet. were restricted to the vicinity of vent mussel assemblages at the Eiffel Tower and Montsegur edifices (see Fig. 2b in^57^). It remains unclear if the differences in end-member chemistry can explain zoanthid absence from the SWC area^23^, as a result of a possible lack of tolerance to particular chemical concentrations. Alternatively, the presence of large assemblages could be facilitated by the availability of larger areas of stabler substrata located in low exposure areas at the base and peripheral zones in the SE area. High supply of food derived from the large mussel assemblages could also contribute to zoanthid success in the SE area. Dense zoanthid aggregations have been detected in other vent peripheries, like at the Tiancheng vent field in the Indian Ocean and the Tow Cam vent field in the south-west Pacific Ocean^11,58^, although their small size (~ 1 cm) could explain their scarcity from imagery observations. The presence of dense clusters of zoanthids provides an opportunity to investigate the adaptations and energy transfer pathways from chemosynthetic environments with high primary productivity to Hexacorallian specimens, including chemoautotrophic microbial associations recently highlighted in octocorals and actiniarians^59,60^.

It is well established that the toxicity of vent fluids excludes species that are not adapted to such conditions, resulting in a marked shift in community composition at the outer periphery of venting area^4^. In our study, this shift was observed within 20 m from venting activity. Beyond that point, megafaunal community was characterised by ~ 30 morphotypes dominated either by deep-sea shrimps or by actiniarians, alcyonaceans and poriferans. Peripheral community composition was not sensitive to differences in the end-member fluid composition between the SE and SWC areas^23^, which is consistent with the similar composition observed in macrofaunal communities across Lucky Strike^61^. Overall, differences in vent fluid composition should become negligible as the vent fluid dilutes in the water column, as suggested by similar chemical concentrations in diffuse vent fluid between the White Castle (SWC) and the Eiffel Tower (SE) edifices. However, the spatial distance from venting activity was associated with a negative correlation of sessile fauna density (i.e., porifera and cnidaria), probably linked to the decreasing stressing conditions as vent exposure declines^5,62–64^. An opposite trend was observed for mobile taxa, like shrimps, possibly feeding opportunistically on organic matter derived from vent primary productivity^17,65^. This not only demonstrates that hydrothermal outflows create strong metre-scale zonation beyond areas of vent emission, but also that species react differently to stressing conditions due to specific functional traits (e.g., mobility) and physiological tolerance^12,64^.

Outside venting areas, substrata and vent exposure strongly influence the distribution of sessile organisms which is a pattern observed in vent fields of the Pacific and Indian oceans^12,17^. Firstly, hard substratum (i.e. slab and basalt) hosted significantly higher faunal density and diversity than the soft volcaniclastic sediments. Soft sediment, typically building up around active vents^8^, can have a negative influence on the sessile fauna since most species require a stable and hard substratum for sustainable anchorage^66^. Secondly, the basaltic habitat supported higher densities of suspension feeders including—blue octocorals and Hexactinellid glass sponges, and carnivorous Cladorhizids—but not necessarily a higher diversity. Basaltic substrata have a boulder-like geomorphology that is associated with higher topographic complexity than the slab, potentially contributing to enhance local hydrodynamics and exposure to food particles, as inferred at other vent fields^11,12^ and other environments such as seamounts or submarine canyons^67,68^, while providing a shelter from direct vent exposure^66^. While not evaluated in this study, bottom currents can interact with terrain orientation and topography affecting the extent of vent influence^24^ and plume exposure in a more complex way than just the distance from active venting. This process could account for the higher faunal density and diversity observed in basaltic areas. But because of the dynamic nature of current speed and direction, computing cumulative exposure to vents requires numerical modelling as well as the integration of oceanographic, geophysical and geochemical processes^69^. Such an approach could bring valuable insight in processes affecting the distribution of peripheral fauna across the various habitats.

### Implications for vent ecology and conservation

As this study is limited by spatial coverage and image resolution, diversity indices are likely underestimated. Expanded imaging across a broader range of environmental conditions is needed to better evaluate the spatial extent of hydrothermal influence at Lucky Strike^70^. Habitat diversity and inactive hydrothermal features should also be considered to obtain a more complete catalogue of benthic megafauna as rare morphotypes remained commonly observed^12,13,71^. Limited imaging coverage (up to 120 m from vents) may underestimate distal vent influence, which can be detected up to a kilometre via chemosynthesis-derived material^8,9^. In addition, image-based classification introduces taxonomic uncertainty, further underestimating biodiversity^72^, especially for peripheral fauna historically less described than iconic vent species^73^. Sampling to improve species description in peripheral areas is essential to robustly identify zonation patterns and standardise community comparisons.

Despite inherent limitations, our imagery sampling was designed specifically to disentangle the relative importance of a combination of independent variables forming a mosaic of habitats structuring communities in and around hydrothermal vents. We observed that biological diversity is influenced by the fine scales (10–20 m) of habitat heterogeneity shaped by interacting variables, like environmental gradients (e.g., vent exposure), habitat suitability that conditions settlement (e.g., substratum hardness) and food exposure (e.g., topographic complexity), in addition to the sporadic presence of keystone structures like large, consolidated and possibly, mature vent edifices. Our study also underlined specific distribution patterns, supposedly influenced by their ecological traits (such as mobility or feeding strategy) which ultimately drives differences in community structure^64^. The diversity of environmental drivers and interactions, along with faunal responses, creates a patchwork of habitats driving the observed mosaic distribution of vent and peripheral assemblages.

This study demonstrated how biodiversity at a hydrothermal vent field is maintained and even enhanced by multiscale processes. Interactions of non-linear processes (e.g., vent fluid dilution, substratum distribution) also pointed out the complexity of mapping the fine-scale mosaic of habitats, that characterises the sphere of hydrothermal influence^70^. Although the development of autonomous underwater vehicles holds potential for extensive and multiscale imagery^74,75^, the fine-scale mosaics of communities present challenges for the establishment of area-based conservation strategies to manage anthropogenic activities like deep-sea mining. One example is the monitoring of an impacted zone before and after disturbance compared to a preserved one^76,77^. Some authors expressed concerns about our limited ability to identify a reference zone that accurately represents the impacted area, due to the patchiness and heterogeneity of the habitat along with the presence of sporadic keystone structures^16,78,79^. In fact, these characteristics contribute to the uniqueness of faunal communities at and around active vents, which we showed to vary at decametre scales. Hence, characterising the spatial structure to define areas of interest for management requires large-scale sampling effort, along-side high-resolution approaches to capture the full range of biological and geological featured over the vent field (~ km^2^) and to match the spatial extent of a commercial mining disturbance.

Currently, planning of seafloor massive sulphide (SMS) mining considers extraction in inactive areas to avoid direct damage to vent-endemic fauna. Therefore, particular attention should be drawn to filling the gap in ecological knowledge of these SMS communities. Our observations complement those made in the nascent body of literature on inactive vent ecology, which increasingly demonstrates higher biodiversity levels with compositionally distinct—and in some cases unique—communities occurring at inactive and peripheral vent habitats, despite limited taxonomic resolution to date^12,16,79^. Our results complement growing scientific evidence that does not support the assumption of inactive hydrothermal vents representing low-biodiversity or ecologically marginal systems. Instead, inactive vents host diverse assemblages potentially sustained by residual chemosynthetic processes and characterized by species compositions distinct from those at active vents. As such, proposals that prioritize inactive vent fields for mineral extraction cannot be justified by assuming a reduced ecological value given their high biodiversity, specific ecological functions, and potential uniqueness^70,79^. Failure to acknowledge this would lead to risk irreversible loss of poorly known but ecologically significant deep-sea communities.

## Supplementary Information

Below is the link to the electronic supplementary material.

Supplementary Material 1

Supplementary Material 2

## Data Availability

Raw images and all annotation data are openly accessible under the DOIs [10.17882/95015](https:/doi.org/10.17882/95015) and [10.17882/106554](https:/doi.org/10.17882/106554) on SEANOE. Spatial distribution of active assemblages are provided in Supplementary Material. R scripts and processed data to compute ecological analyses and Tukey *post hoc* test results are supplemented to this paper.
