# Supplementary material for: The role of habitat mosaics on biological communities at hydrothermal vents and their periphery
Source: Sci Rep. 2026 Feb 18;16:9751. doi: 10.1038/s41598-026-39544-x (PMC13013592; doi:10.1038/s41598-026-39544-x)
Supplement: Supplementary file 1 — Supplementary Material 1 [file 41598_2026_39544_MOESM1_ESM.docx]

Supplementary Material to **The Role of Habitat Mosaics on Biological Communities at Hydrothermal Vents and Their Periphery**

Van Audenhaege Loïc^1,2^*, Sarrazin Jozée^2^, Ramière Annah^2^, Borremans Catherine^2^, Marcillat Marin^2^, Soto Vega Pedro Juan^2^, Cannat Mathilde^3^, Marticorena Julien^4^, Colaço Ana^5^, Matabos Marjolaine^2^

*^1^ National Oceanography Centre, Southampton, United Kingdom*

*^2^ Univ Brest, Ifremer, BEEP, F-29280 Plouzané, France*

*^3^ Université Paris Cité, Institut de Physique du Globe de Paris, UMR 7154 CNRS, Paris, France*

*^4^ ABYSSA, French Company for the Deep-Ocean Exploration, Anglet, France*

*^5^ University of the Azores, Institute of Marine Sciences – OKEANOS, Horta, Portugal*

*Corresponding author: [loic.vanaudenhaege@gmail.com/loicva@noc.ac.uk](mailto:loic.vanaudenhaege@gmail.com/loicva@noc.ac.uk)


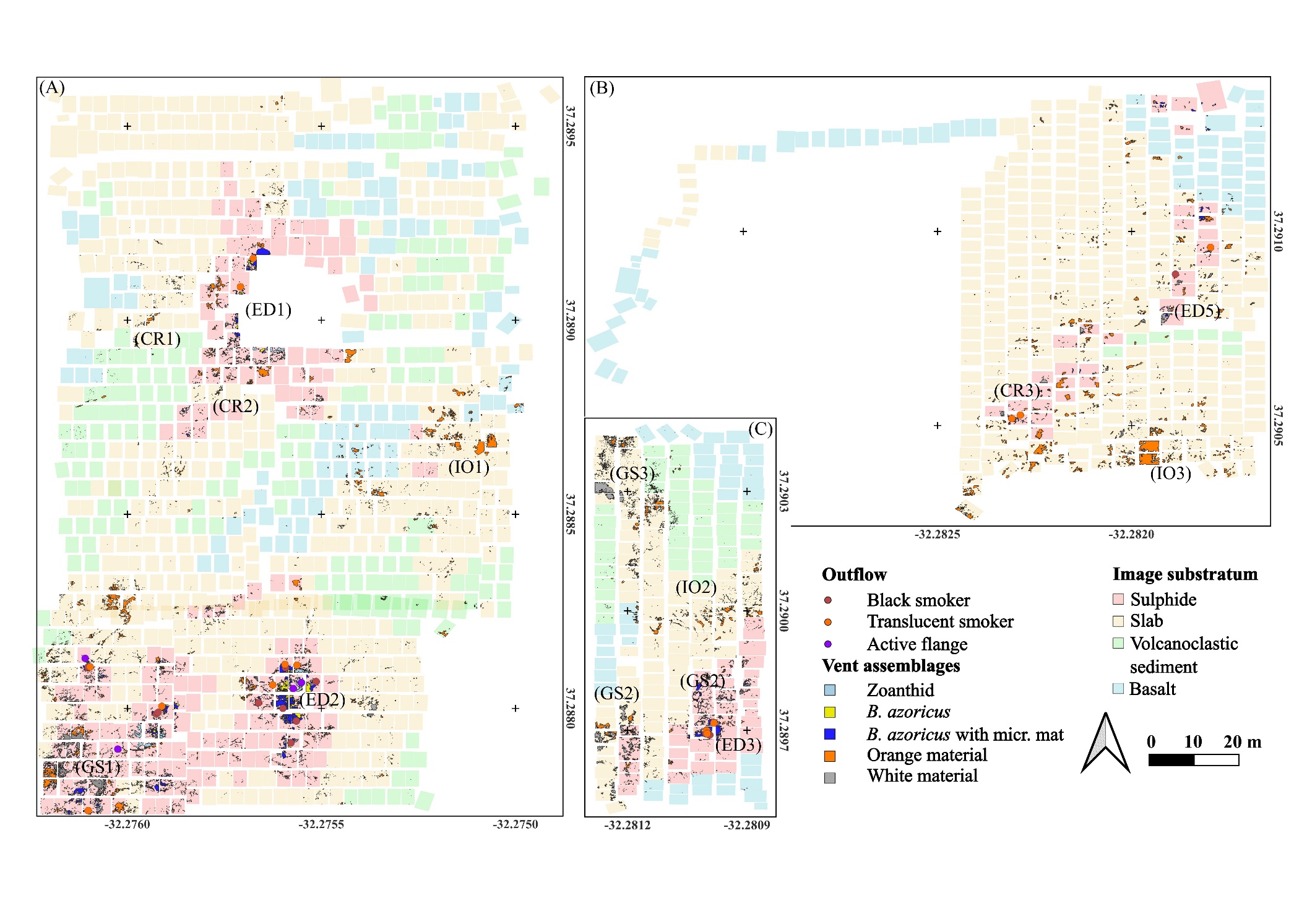
 Supplementary Figure 1 – Maps of distribution of hydrothermal features and substrata for the sites in the south-east (A) including Eiffel Tower in the north and Montségur in the south-east, and in the south-west centre including (B) Sapins and (C) White Castle. Image substrata are plot with a colour code associated with the imprint of each image annotated. Outflows and flanges are located with colour-specific points. To ease presentation of the results, the presence of different types of hydrothermal settings is presented with a character code: ED = Main edifice, GS = Grey sandy areas, CR = Cracks in the periphery of the main edifice and IO = isolated orange material. Vent assemblages are shown with polygons of different colours. The coordinate grid is expressed in WGS84. Note the gap in A showing the location of the Eiffel Tower edifice, a structure ~ 11 m high that was not possible to image with a downward-looking camera configuration.
